# SREBP Controls Oxygen-Dependent Mobilization of Retrotransposons in Fission Yeast

**DOI:** 10.1371/journal.pgen.0030131

**Published:** 2007-08-10

**Authors:** Alfica Sehgal, Chih-Yung S Lee, Peter J Espenshade

**Affiliations:** Department of Cell Biology, Johns Hopkins University School of Medicine, Baltimore, Maryland, Unites States of America; Fred Hutchinson Cancer Research Center, United States of America

## Abstract

Retrotransposons are mobile genetic elements that proliferate through an RNA intermediate. Transposons do not encode transcription factors and thus rely on host factors for mRNA expression and survival. Despite information regarding conditions under which elements are upregulated, much remains to be learned about the regulatory mechanisms or factors controlling retrotransposon expression. Here, we report that low oxygen activates the fission yeast Tf2 family of retrotransposons. Sre1, the yeast ortholog of the mammalian membrane-bound transcription factor sterol regulatory element binding protein (SREBP), directly induces the expression and mobilization of Tf2 retrotransposons under low oxygen. Sre1 binds to DNA sequences in the Tf2 long terminal repeat that functions as an oxygen-dependent promoter. We find that Tf2 solo long terminal repeats throughout the genome direct oxygen-dependent expression of adjacent coding and noncoding sequences, providing a potential mechanism for the generation of oxygen-dependent gene expression.

## Author Summary

Transposons are present at high copy number in diverse organisms ranging from single-celled bacteria to complex mammals and plants. Transposons are mobile genetic elements that can replicate and move to new locations within the genome. An ongoing debate exists regarding whether transposons are merely genetic parasites or whether they confer a benefit to the host organism. Previous studies have demonstrated that mobilization of transposable elements is induced in response to different cell stresses. Here, we describe the direct mechanism by which the fission yeast Tf2 class of retrotransposons is physiologically activated in response to changes in environmental oxygen. Tf2 transcription and mobilization are dramatically induced under low oxygen by the yeast ortholog of mammalian SREBP, a transcription factor that controls cholesterol homeostasis. Our studies demonstrate that Tf2 elements direct oxygen-dependent transcription of adjacent sequences, and a genome-wide survey identified several genes whose expression is under Tf2 control. These findings suggest that mobilization of Tf2 to new locations in the genome could reengineer the cell's transcriptional network with potentially beneficial consequences to the host.

## Introduction

Transposable elements are mobile DNA sequences that are present in most eukaryotic organisms and occupy a large fraction of sequenced genomes; for example, human (∼50%) [1], C. elegans (12%) [2], and plants (10%–80%) [3]. Once viewed as simple mutagens, transposable elements are increasingly seen as playing a significant role in evolution by affecting genome size, structure, and host gene expression [4]. Retrotransposons are genetic elements that propagate through reverse transcription of their RNA and integration of the resulting cDNA into another genomic locus [4]. Retrotransposons resemble retroviruses in both gene structure and replication mechanism, but lack a viral envelope protein required for cell–cell infectivity. Long terminal repeat (LTR) retrotransposons have terminally redundant ends flanking an internal coding region that codes for viral particle coat, reverse transcriptase, integrase, and protease. The LTR is the functional promoter for the transposon and can be divided into three regions U3, R, and U5. U3 contains *cis-*acting sequences involved in transcriptional regulation, R encodes the transcription initiation site, and U5 carries the transcriptional termination signal, which plays a role in the 3′ LTR [5]. In addition to full-length retrotransposons, genomes contain many solitary LTRs and fragments of retrotransposons. Solo LTRs are footprints of previously intact retrotransposons and can be generated by intra-element homologous recombination between LTRs. Thus, solo LTRs can function as promoters, influencing transcription of adjacent genes. Several examples of genes transcribed by a solo LTR from a human endogenous retrovirus have been found: apolipoprotein-C1, endothelin-B receptor [6], β-globin [7], and *Mid1* [8].

Transposable elements can also negatively impact the genome by causing mutations and affecting host viability, necessitating that a balance exist between element expansion and host mutagenesis [9]. To control this balance, mechanisms exist that limit expression of retrotransposons including RNAi, heterochromatization, cosuppression, dependence on host factors, and regulated element transcription [3,10–13]. Regulated transcriptional control provides a mechanism for restricting element expression to defined cellular conditions. Retrotransposon transcription and subsequent transposition are upregulated by different environmental conditions and stresses. Ionizing radiation, DNA damage, mating pheromone, and nutrient limitation activate S. cerevisiae Ty elements [10,14,15]; heat and sodium azide induce *Drosophila* copia elements [16]; and wounding, biotic elicitors, and pathogen attack activate Tnt1 in the Solanaceae plant family [17]. One well-understood regulatory mechanism involves the transcriptional activators Ste12 and Tec1, which regulate S. cerevisiae Ty1 elements in response to mating pheromones, nitrogen starvation, and invasive growth signals [18–20]. Transcription regulatory sequences for Ty1 exist both in the 5′ LTR and the element open reading frames.

Here, we report the physiological induction of the fission yeast Tf2 transposon family by low oxygen and describe the mechanism of this regulated expression. Schizosaccharomyces pombe contains two families of retrotransposons, Tf1 and Tf2; however, the laboratory-adapted strain has lost Tf1 [21]. Tf2 transposons are 4.9 kb in length and are flanked by 349-bp LTRs. When overexpressed from a heterologous promoter, Tf2 retrotransposons show a low transposition frequency, preferring to recombine with existing transposons [22,23]. While much is known about the mechanism of Tf retrotransposition in fission yeast, little is known about the regulation of element transcription. Genome-wide analyses indicate that neither heterochromatization nor RNAi-mediated silencing plays a major role in regulation of Tf2 element expression [24,25]. We previously identified Tf2 transposons as targets of the oxygen-dependent transcription factor Sre1 [26]. Sre1 is the fission yeast ortholog of sterol regulatory element binding protein (SREBP) that controls lipid homeostasis in mammalian cells [27–29]. Under low oxygen, Sre1 is proteolytically processed, enters the nucleus, and activates genes required for adaptation to low oxygen growth [26]. Here, we demonstrate that Sre1 directly activates Tf2 transcription and cDNA mobilization under low oxygen by binding to sequences in the Tf2 LTR. Sre1 does not activate only a single element but rather the family as a whole. Interestingly, Tf2 solo LTRs function as oxygen-dependent promoters that are capable of directing low oxygen transcription of adjacent DNA sequences. Taken together, these studies describe a detailed mechanism for the transcriptional regulation of retrotransposons by low oxygen, reveal a new environmental condition for element mobilization, and provide an example of LTR-mediated transcriptional control of host gene expression.

## Results/Discussion

**Figure 1:**
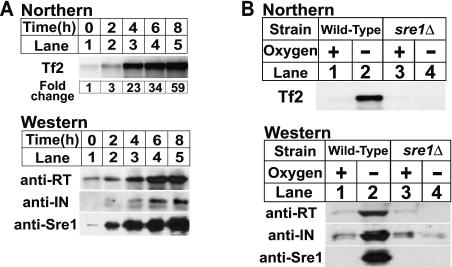
Low Oxygen Expression of Tf2 mRNA and Protein Requires Sre1 (A) Wild-type yeast were cultured in the absence of oxygen for increasing time. Upper panel: total RNA (10 μg) was subjected to northern analysis using a Tf2 probe. Lower panel: cell extracts (40 μg) were analyzed by immunoblotting using antibodies to Tf2 reverse transcriptase (RT), integrase (IN), and the nuclear form of Sre1 [23,27]. (B) Wild-type and *sre1*Δ yeast were cultured +/− oxygen for 6 h and processed for northern and immunoblot analysis.

We identified Tf2 transposons as targets of the oxygen-dependent transcription factor Sre1 in a global analysis of low-oxygen gene expression. [26]. To confirm that Tf2 transcription was induced under low oxygen and required Sre1, we analyzed expression of Tf2 mRNA and Tf2 encoded proteins from cells grown in the presence or absence of oxygen (Figure 1A, upper panel). Tf2 mRNA increased after shifting to low oxygen, reaching a level 59-fold higher than in the presence of oxygen after 8 h. Expression of Tf2 reverse transcriptase and integrase protein was similarly induced (Figure 1A, lower panels). Consistent with our microarray data [26], Tf2 mRNA and protein expression were not induced by low oxygen in a *sre1*Δ strain (Figure 1B). Thus, Sre1 is required for the low oxygen induction of Tf2 retrotransposon mRNA and protein.

**Figure 2:**
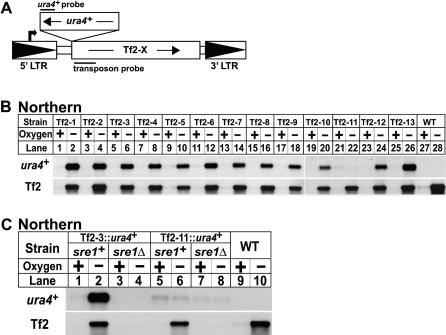
Sre1 Controls Low Oxygen Expression of Multiple Tf2 Elements (A) Scheme for tagging individual Tf2 elements with *ura4^+^*. Bold arrow denotes Tf2 transcription initiation. Northern probe positions are indicated. (B) Yeast containing a single Tf2 element tagged by *ura4^+^* or wild-type yeast were grown +/− oxygen for 8 h and processed for northern analysis using strand-specific DNA probes to detect either the tagged Tf2 element (*ura4^+^* probe) or all 13 Tf2 elements (Tf2 probe). (C) Wild-type or *sre1*Δ yeast containing a tagged Tf2 element were grown +/− oxygen for 6 h and processed for northern analysis. All Tf2 elements tested (Tf2–4, Tf2–7, and Tf2–10) gave results identical to Tf2–3.

Fission yeast contains 13 full-length Tf2 transposons whose coding sequences are 99% identical at the DNA level and thus cannot be distinguished by hybridization [30]. To analyze the expression of individual transposons, we designed a strategy to tag each transposon with *ura4^+^* (Figure 2A). We generated 13 different strains (Tf2–1 to Tf2–13), each carrying a single, tagged Tf2 element. These 13 strains and an untagged wild-type strain were grown in the presence or absence of oxygen for 8 h and processed for northern analysis using a strand-specific *ura4* ^+^ probe. Expression of 12 out of 13 transposons increased under low oxygen (Figure 2B, upper panel). Notably, Tf2–11 was not induced despite low oxygen expression of other Tf2 elements in this strain (Figure 2B, lanes 21–22). Hereafter, we refer to the 12 coregulated elements collectively as Tf2. To test if low oxygen induction required Sre1, we deleted *sre1^+^* from five Tf2-*ura4^+^* tagged strains (Tf2–3, 4, 7, 10, and 11). Low oxygen induction of Tf2 transposons required Sre1 (Figure 2C), while deletion of *sre1^+^* had no effect on Tf2–11 expression. These data demonstrate that with the exception of Tf2–11, Sre1 controls low oxygen expression of Tf2 transposons.

**Figure 3:**
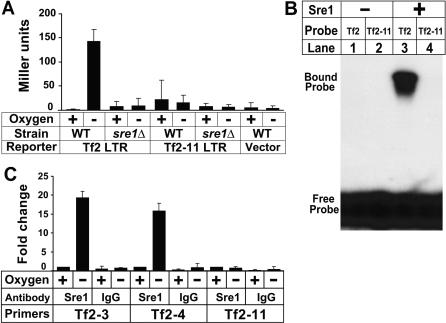
Tf2 LTR Is an Oxygen-Dependent Promoter (A) Wild-type and *sre1*Δ yeast carrying Tf2 LTR-*lacZ*, Tf2–11 LTR-*lacZ*, or *lacZ* reporter plasmids were grown +/− oxygen for 6 h and assayed for β-galactosidase activity [26]. Error bars denote one standard deviation among three biological replicates. (B) Sre1 DNA binding domain (aa 256–366) was incubated with indicated ^32^P-labeled DNA probes and subjected to electrophoretic mobility shift assay [26]. (C) Wild-type yeast were grown +/− oxygen for 6 h and subjected to chromatin immunoprecipitation using anti-Sre1 IgG or rabbit IgG. Bound DNA was normalized to wild type + oxygen for each primer pair. The fraction of bound DNA values for the pulldown with anti-Sre1 under aerobic conditions were 0.0027 (Tf2–3), 0.003 (Tf2–4), and 0.017 (Tf2–11). Error bars denote one standard deviation among three experimental replicates.

Tf2 transcription initiates in the 5′ LTR [31]. To test if the Tf2 LTR is sufficient to promote oxygen-dependent gene expression, we assayed expression of a *lacZ* reporter driven by either Tf2 LTR or Tf2–11 LTR in the presence or absence of oxygen (Figure 3A). Expression of Tf2 LTR-*lacZ* was induced more than 100-fold in the absence of oxygen and this induction required *sre1^+^*. Cells carrying Tf2–11 LTR-*lacZ* showed a background level of β-galactosidase activity that was not regulated by oxygen or *sre1^+^*. A search of the Tf2 5′ LTR revealed a DNA sequence (5′-ATCGTACCAT-3′) located 443 bp upstream of the Tf2 ORF in 12 elements that fits the consensus for a Sre1 regulatory element (SRE) [26]. Importantly, this sequence was different in Tf2–11 (5′-ATCGTAGATA-3′) and did not match the SRE consensus (Figure S1). In vitro DNA binding assays confirmed that Sre1 bound to the Tf2 SRE sequence, but not to the sequence present in Tf2–11 (Figure 3B). Furthermore, chromatin immunoprecipitation experiments demonstrated that Sre1 bound to Tf2 LTR in vivo under low oxygen conditions, but not to Tf2–11 LTR (Figure 3C). Thus, Tf2–11 contains a natural mutation in the Sre1 DNA binding sequence that prevents low oxygen regulation of this transposon [30]. Collectively, these data demonstrate that Tf2 LTR functions as an oxygen-dependent promoter that is directly regulated by Sre1.

Given that low oxygen induced expression of Tf2 mRNA and protein, we next tested whether induction of Tf2 by Sre1 resulted in increased element transposition. Previous studies examined fission yeast Tf1 or Tf2 mobilization when these elements were highly overexpressed in the presence of oxygen from a heterologous promoter [23,32,33]. Interestingly unlike Tf1, the majority of Tf2 mobilization events (>70%) did not require the Tf2 integrase and thus occurred by cDNA recombination [23]. Here, we measured the ability of an endogenous Tf2 element to mobilize in response to changes in environmental oxygen. To monitor transposition, we inserted an intron-containing neomycin resistance gene into the Tf2–12 3′ UTR in the opposite orientation to the Tf2–12 ORF (Figure S2). This neomycin resistance gene was interrupted by an artificial intron *(neoAI)* in the antisense direction that is spliced out of the Tf2–12 mRNA [34]. In this way, cells become G418 resistant only when Tf2–12 has mobilized and inserted into the genome via a cDNA intermediate.

**Figure 4:**
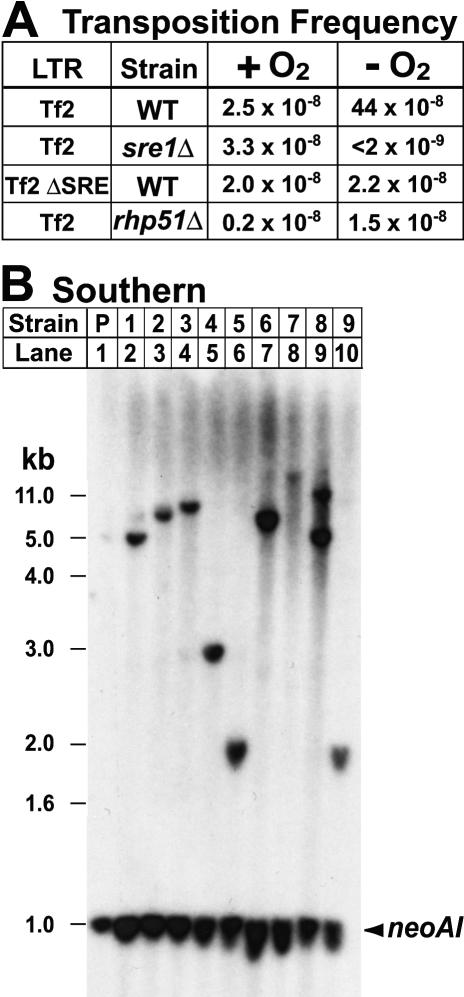
Low Oxygen Stimulates Sre1-Dependent, Tf2 Mobilization (A) Tf2–12-*neoAI* yeast cells were grown in the absence of oxygen for 8 h to induce transcription, cDNA synthesis, and mobilization. Cells were assayed for acquisition of G418-resistance as described in Materials and Methods. Frequency of G418-resistance for different strains is shown. (B) Genomic DNA was isolated from the parent Tf2–12-*neoAI* strain (lane 1) and nine G418-resistant progeny strains (lanes 2–10), digested with HindIII, and analyzed by Southern blotting using a *neo* probe. *neoAI* denotes the *neo* gene with the artificial intron present in the Tf2–12-*neoAI* parent strain.

To measure transposition, we cultured Tf2–12-*neoAI* cells in the presence or absence of oxygen for 8 h and then plated cells on selective medium containing G418 to determine the frequency of transposition. The basal aerobic frequency of Tf2–12-*neoAI* transposition (2.5 × 10^−8^/cell) increased 18-fold (44 × 10^−8^/cell) under low oxygen (Figure 4A). As expected, this oxygen-dependent increase in transposition frequency required Sre1. First, cells lacking *sre1^+^* showed no increase in transposition under low oxygen. Second, deletion of the Sre1 DNA binding sequence from the Tf2 5′ LTR resulted in the loss of oxygen-dependent induction of transposition (Tf2 ΔSRE, Figure 4A). Importantly, this assay monitored only one of the 12 oxygen-responsive Tf2 elements in the fission yeast genome [30]. Thus, we expect the actual transposition frequency under low oxygen to be >10-fold higher (∼5 × 10^−6^/cell).

Southern blot analysis for the *neo* gene in nine independent G418-resistant colonies derived from a low oxygen culture revealed that each strain contained at least one novel Tf2–12-*neo* insertion not present in the parent strain (Figure 4B). Using a combination of Southern blotting, PCR-based screening, and ligation-mediated PCR, we determined the location of the spliced Tf2–12-*neo* cassette in 20 randomly selected low oxygen clones (22 total insertion events). All of the insertion events appeared to result from homologous recombination of Tf2 cDNA. By identifying sequences downstream of the Tf2–12-*neo* 3′ LTR, we determined that 12 insertion events occurred upstream of an existing Tf2 resulting in tandem Tf2 elements. Since S. pombe contains two tandem transposons (Tf2–8 and Tf2–7), these 12 events represent either a replacement of Tf2–8 or a new insertion upstream of an existing Tf2 element. In the remaining ten events, the Tf2–12-*neo* cDNA replaced an existing Tf2 element: Tf2–1 (two events), Tf2–7 (one event), Tf2–9 (one event), Tf2–10 (one event), Tf2–11 (one event), and Tf2–12 (four events). Replacements may occur preferentially at Tf2–12 due to the additional homology that exists between Tf2–12-*neoAI* and the Tf2–12-*neo* cDNA as compared to other Tf2 elements.

To test directly whether recombination was required for Tf2 mobilization, we determined the transposition frequency of Tf2–12-*neo* in cells lacking *rhp51^+^*, which is required for homologous recombination in S. pombe [35]. The Tf2–12-*neo* transposition frequency under low oxygen decreased 29-fold in *rhp51*Δ strain, and mobilization remained oxygen-dependent (Figure 4A). This decrease in transposition frequency indicated that Tf2 mobilization requires homologous recombination and was consistent with our mapping of these Tf2–12-*neo* cDNA insertions (*n* = 6) to existing Tf2 loci. In addition, the Tf2-*neo* insertions lacked new target site duplications flanking the elements, which are characteristic of integrase-mediated insertion events. Based on the high amino acid sequence identity among the 12 copies of Tf2 (99%), we infer that other Tf2 elements mobilize by recombination. A similar mechanism of cDNA mobilization has been observed for Ty elements in S. cerevisiae [36,37]. Mobilization of Tf2 by cDNA recombination to existing elements has been termed “integration site recycling” and may serve as a mechanism to protect the host cell genome while allowing Tf2 elements to evolve [23]. Collectively, these data demonstrate that Sre1 induces mobilization of Tf2 retrotransposons by homologous recombination in response to low oxygen.

**Figure 5:**
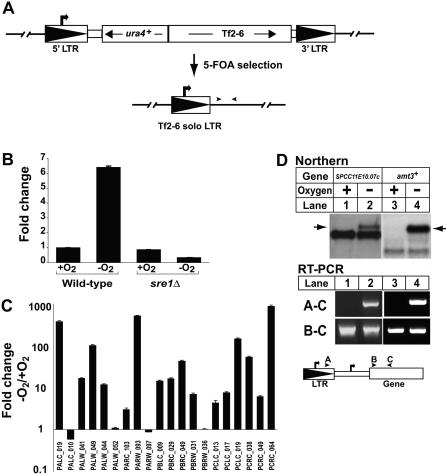
Endogenous Solo LTRs Function as Oxygen-Dependent Promoters (A) Scheme for generating Tf2–6 solo LTR. Recombination between intra-element LTRs yields solo LTR. Arrowheads denote position of RT-PCR primers. (B) Wild-type and *sre1*Δ yeast containing Tf2–6 solo LTR were cultured +/− oxygen for 4 h. Transcription downstream of the LTR was quantified by real-time RT-PCR and normalized to values for wild-type cells + oxygen. Error bars denote standard deviation among three real-time RT-PCR replicates. (C) Wild-type yeast were cultured +/− oxygen for 4 h. Transcription of sequences ∼100 bp downstream of 20 different Tf2 solo LTRs was quantified by real-time RT-PCR as described in Materials and Methods. Fold change in transcription after shifting to low oxygen is shown. Error bars denote standard deviation among three real-time RT-PCR replicates. (D) Wild-type yeast were cultured +/− oxygen for 6 h. Upper panel: Northern analysis using *SPCC11E10.07c* and *SPAC2E1P3.02c* probes. Arrows indicate Tf2 LTR derived transcript. Lower panel: RT-PCR to detect transcripts originating either in Tf2 LTR (A–C) or the full mRNA transcripts (B–C). Bold arrows denote transcription initiation sites. Arrowheads denote primer positions.

The presence of 13 Tf2 elements and 35 Tf2 solo LTRs in the S. pombe genome suggests that a small fraction of mobilization events occur at new positions in the genome [30]. Unlike most characterized retrotransposons [38], fission yeast Tf elements preferentially insert upstream (∼100–400 bp) of RNA polymerase II transcribed genes [30,32,33]. Together with our data, this insertion site bias for RNA polymerase II promoters suggested that Sre1 may control low oxygen expression of genes adjacent to solo LTRs. To test this, we took advantage of the fact that solo LTRs can be formed by homologous recombination. We generated a new Tf2 solo LTR from the *ura4* ^+^-tagged Tf2–6 element by counterselecting for expression of *ura4^+^* on medium containing 5-fluoroorotic acid (Figure 5A) [39]. Next, we tested the ability of Tf2–6 solo LTR to promote transcription of adjacent sequences using quantitative RT-PCR and primers adjacent to the LTR. We detected Sre1-dependent, oxygen-dependent transcription downstream of Tf2–6 solo LTR, demonstrating that solo LTRs can direct transcription of non-Tf2 sequences (Figure 5B).

To test the promoter capabilities of preexisting solo LTRs in the fission yeast genome, we identified 25 solo LTRs that resembled Tf2 LTR and contained an intact SRE [30]. Using primers to adjacent noncoding downstream sequences, we detected transcripts from 20 solo LTRs by real-time reverse-transcriptase PCR (RT-PCR), and expression of 16 out of 20 transcripts was oxygen dependent (Figure 5C). Transcripts from four solo LTRs were not regulated by oxygen, possibly due to local chromatin effects, stability of LTR-specific transcripts, or the position of the amplifying primers. These results demonstrate that solo LTRs scattered across the S. pombe genome are functional oxygen-dependent promoters.

To investigate the genome-wide impact of Tf2 LTRs on low oxygen gene expression, we designed an experiment to identify RNA transcripts containing Tf2 LTR sequences using a S. pombe genomic tiling array. Briefly, we amplified cDNA from wild-type cells grown under low oxygen using a Tf2 LTR-specific forward primer and a random reverse primer (Figure S3; Materials and Methods). The amplified DNA was labeled and used to probe the Affymetrix S. pombe genome tiling array. The labeled probes should identify regions of the genome encoded in Tf2 LTR-containing RNAs. To eliminate artifacts due to nonspecific LTR primer binding, two tiling array experiments were performed using two different Tf2 LTR forward primers. The sequences presented here were identified in both experiments. As expected, we identified each of the Tf2 transposons, validating our methodology. Tf2–11 was also identified, presumably due to its basal expression (Figure 2C) or to cross-hybridization with probes from other Tf2 elements.

Importantly, the tiling experiment also identified four additional open reading frames (*SPCC11E10.07c, SPAC1B3.08, SPAC823.14,* and *SPAC2E1P3.02c*) using our cut-off criteria (*p* < 0.05), and each of these four genes were positioned downstream of a Tf2 LTR. Using RT-PCR and gene-specific primers, we confirmed that each gene was encoded in an oxygen-dependent transcript that originated from a Tf2 LTR. Northern analysis for the first gene *SPCC11E10.07c*, which codes for the alpha subunit of the translation initiation factor eIF2B, detected a novel oxygen-dependent transcript (Figure 5D, arrow upper panel). This low oxygen transcript represented 19% of the total message and originated from the upstream solo Tf2 LTR as confirmed by RT-PCR (Figure 5D, lower panel). The solo LTR for *SPCC11E10.07c* corresponds to PCRC_038 in Figure 5C, which showed a 55-fold increase in expression under low oxygen. Interestingly, *GCN3,* the S. cerevisiae homolog of *SPCC11E10.07c*, has a Ty1 solo LTR positioned 499 bp upstream.

Northern blot analysis for the second gene *SPAC1B3.08* showed a pattern similar to *SPCC11E10.07c* with an oxygen-dependent upper transcript representing 16% of the total (unpublished data). The corresponding upstream solo LTR for *SPAC1B3.08* is PARW_093 in Figure 5C. For the third gene *SPAC823.14*, we confirmed an LTR-derived low oxygen transcript originating from the upstream solo LTR PALW_049 by RT-PCR (Figure 5C). However, we were unable to detect a novel low oxygen transcript for *SPAC823.14* by northern blotting*,* possibly because of the lower sensitivity of northern analysis. Finally, *amt3^+^/SPAC2E1P3.02c*, which encodes ammonium transporter 3, is positioned downstream of the Tf2–3 element and not a solo-LTR. Northern analysis revealed a longer, major transcript under low oxygen, which accounts for 78% of the total low oxygen transcript (Figure 5D, arrow upper panel). RT-PCR analysis confirmed that this upper transcript originated in the Tf2–3 LTR. Deletion of the solo LTR PCRC_038 and the Tf2–3 element resulted in the loss of the upper oxygen-dependent transcripts for *SPCC11E10.07c* and *amt3^+^*, respectively, consistent with transcription initiating within the Tf2 LTR (unpublished data). The functional significance of these oxygen-dependent, LTR-derived transcripts remains to be determined. In addition to the examples mentioned above for open reading frames, we detected LTR-derived transcripts from many of the solo LTRs examined in Figure 5C. However, these transcripts had low signal intensity and did not make our cut-off, possibly due to increased turnover of these noncoding RNAs. Together, these data establish Tf2 LTRs as promoters that can direct oxygen-dependent transcription of adjacent genes, demonstrating the ability of Tf2 transposons to regulate the S. pombe transcriptome.

Retrotransposons in different organisms have been shown to respond to a variety of environmental signals and stresses [10,40]. We report that the hypoxic transcription factor Sre1 directly controls the low oxygen induction of Tf2 retrotransposon expression and mobilization. In a genome-wide transcriptional analysis of environmental stress responses, Tf2 transposons were shown to be upregulated by heat and peroxide stress (∼4-fold), but not by heavy metal, osmotic stress, or a DNA alkylating agent [41]. We observed a similar induction of Tf2 transcription by heat stress, but this upregulation did not require Sre1, suggesting that other factors may regulate Tf2 expression. Peroxide stress also induced Tf2 transcription in a Sre1-dependent manner, consistent with the fact that hydrogen peroxide activates Sre1 (unpublished data). In addition, Tf2 was not upregulated by treatment of cells with the endoplasmic reticulum stress inducer tunicamycin. Thus, Sre1-dependent induction of Tf2 is a specific response, as Tf2 transcription is not broadly affected by environmental stress.

Accumulating evidence implicates transposable elements as regulators of gene expression in eukaryotes as diverse as plants and humans [42,43]. Transposable elements contribute to genomic evolution by donating regulatory elements, providing alternative promoters, or causing mutations by inserting into genes [44]. Our results now provide evidence for regulation of endogenous gene expression by transposons in fission yeast. Here, we report the regulation of Tf2 retrotransposons by oxygen and demonstrate that Tf2 LTRs direct low oxygen transcription of adjacent coding sequences. Given that the Tf family of transposons insert upstream of RNA polymerase II promoters, we speculate that Tf2 insertions may provide a mechanism for generating new oxygen-dependent gene expression.

## Materials and Methods

Yeast strains, media, and standard procedures including northern blotting, western blotting, and β-galactosidase assays have been described previously [26,27]. Table S1 contains sequences of oligonucleotides used.

### Chromosomal tagging of transposons.

To tag individual Tf2 elements, the 1.8-kb *ura4^+^* cassette was inserted upstream of the Tf2 ORF by homologous recombination using standard techniques [45]. The location of *ura4^+^* insertion was confirmed by PCR using unique forward primers designed upstream of each transposon and a common reverse primer in the *ura4^+^* cassette.

To generate the Tf2–6 solo LTR, the *ura4^+^* tagged Tf2–6 strain was plated on Edinburgh minimal medium containing 1 mg/ml 5-fluoroorotic acid at a density of 10^6^ cells per plate. The 5-fluoroorotic acid–resistant colonies were streaked for singles and the absence of Tf2–6 was confirmed by sequencing the PCR product across the transposon. This strain is referred to as Tf2–6 solo LTR.

The *neoAI* tagging of Tf2 and the transposition assay were modified from established protocols [23]. The Tf2–12 *neoAI* was tagged by homologous recombination following transformation of a linear DNA fragment assembled from the following DNA fragments: 670 bp of Tf2–12 (bp 3871–4534), *neoAI* cassette [23], 3′ UTR and 3′ LTR of Tf2–12 (bp 4535–4900), 1.8-kb HindIII *ura4^+^* cassette from pREP4x [46], and the 500 bp downstream of Tf2–12 on the chromosome. These fragments were assembled in pBluescript (Stratagene, http://www.stratagene.com) and the linear fragment used for transformation was released with ApaI and SacI. Transformants obtained on selective medium lacking uracil were screened by PCR and confirmed by Southern blotting [47]. These clones were used as parents for transposition assays. A diagram of the tagged locus is shown in Figure S2.

### Generation of Tf2 ΔSRE strain.

The strain Tf2 ΔSRE contains Tf2–12 *neoAI,* in which the Sre1 DNA binding site (SRE) has been deleted from the 5′ LTR of Tf2–12. Tf2 ΔSRE was made using the Cre-loxP method for marker rescue [48]. Using this technique, a strain was created that contains a deletion of the 10-bp SRE sequence (ATCGTACCAT) in the Tf2–12 5′ LTR (Figure S1) and an insertion upstream of the Tf2–12 5′ LTR consisting of the 34-bp loxP sequence (5′-ATAACTTCGTATAGCATACATTATACGAAGTTAT-3′) and 19 bp of plasmid sequence (5′-CGAAGTTGAATTCCTGCAG-3′). This strain was then transformed with the ApaI-SacI cassette described above to introduce *neoAI* in the 3′ UTR of Tf2–12, resulting in the strain Tf2 ΔSRE.

### Transposition assay.

Tf2–12-*neoAI* yeast cells were cultured in the presence or absence of oxygen for 8 h. Yeast (1 × 10^7^ cells) were plated on rich medium (YES) containing 100 μg/ml G418 [27]. After 16 h, cells were replica plated to a second YES+G418 plate, and G418-resistance was confirmed by retesting individual colonies. Four independently derived Tf2–12-*neoAI* strains were used and the data were pooled. For the Tf2 ΔSRE experiment, two independent strains were used. A total of ∼5 × 10^8^ cells/genotype was scored for mobilization both +/− oxygen. Genomic DNA from these independent G418 resistant clones were digested with HindIII and processed for Southern blotting. To identify the site of integration, a combination of inverse PCR and vectorette PCR was performed. Briefly, for inverse PCR, DNA was digested with restriction enzyme TaqI, self-ligated, and amplified using two divergent oligos positioned in the *neoAI* gene cassette. Vectorette PCR protocol was a modification of previously described methods [49]. Genomic DNA digested with EcoRI or SpeI was used for ligation with vectorette oligos. The PCR products obtained from both of these techniques were cloned using TopoTA (Invitrogen, http://www.invitrogen.com) vector and sequenced.

### Chromatin immunoprecipitation.

Assay was performed as described previously with minor modifications [26]. Binding of Sre1 to Tf2 LTR sequence was quantified by real-time PCR using Tf2 specific LTR primers. Fraction bound was calculated using the formula 2^(Ct_input_^ ^− Ct_pulldown_^^)^ for each treatment. All the values obtained for aerobic samples with anti-Sre1 antibodies were set to one and the other values were normalized accordingly.

### RT-PCR.

cDNA was made from DNAse-treated RNA using Superscript II (Invitrogen). The cDNA used for detecting solo LTR derived transcripts was made with random hexamers. This was then used for real-time PCR amplification using a Bio-Rad iQ Cycler (http://www.bio-rad.com) and SyBr-Green mix from ABgene (http://www.abgene.com). cDNA preparations made without reverse transcriptase were used to control for signal from any contaminating DNA. Oligos to *hcs1^+^* were used to normalize between reactions (*hcs1^+^* transcript levels were unchanged among the tested conditions)[27]. Forward oligos used to detect solo LTR transcripts were positioned ∼20 bp downstream of LTR and yielded ∼100-bp products. To confirm the tiling array candidate transcripts, oligo dT-primed cDNA served as template for PCR with transcript or LTR-specific primers.

### S. pombe genome tiling array.

To detect transcripts containing LTR sequence, cDNA was synthesized using mRNA isolated from yeast cells grown in the absence of oxygen for 8 h and an oligonucleotide containing random hexamers with a 5′ adapter sequence (Figure S3 and Table S1). Following purification, the first-strand cDNA was copied to double-stranded DNA using a LTR-specific oligo and four cycles of amplification. This DNA was amplified for eight cycles using the LTR-specific oligo and the adapter oligo with Platinum Taq (Invitrogen). The resulting amplicons were labeled using Affymetrix cDNA labeling kit (http://www.affymetrix.com) and hybridized to a GeneChip S. pombe Tiling 1.0FR Array using manufacturer's protocols (Affymetrix). This high-density tiling array contains 25-mer oligos for both DNA strands that overlap by 5 bp, giving approximately 20-bp resolution. The data were analyzed using Partek GS software (Partek, http://www.partek.com/). Positive transcripts were identified using a test region of 100 bp, a signal intensity >3.8, and *p*-value <0.05, for continuous signals present over >200-bp regions. Two independent experiments were performed using different, nonoverlapping LTR-specific oligos to control for nonspecific amplification.

## Supporting Information

Figure S1 Tf2 LTR Sequence Alignment Alignment of 5′ LTR DNA sequences from Tf2–3 and Tf2–11. Red overline denotes Sre1 DNA binding site (SRE). Green overline and bold arrow denote TATA box and transcription initiation site, respectively.
(23 KB DOC)

Figure S2 Diagram of Tf2 Mobilization Transcription of Tf2–12 neoAI results in removal of artificial intron from neo. Following reverse transcription and integration, neo gene product is made giving resistance to G418. Hatched area denotes intron.
(875 KB EPS)).

Figure S3 Outline of Synthesis of Labeled DNA Probe for Tiling Array cDNA was synthesized using an oligo with random hexamers and a 5′ adaptor sequence. This cDNA was then amplified for four cycles using only an LTR-specific primer. This DNA product was further amplified using the LTR primer and the adaptor primer for eight cycles. The amplified DNA was labeled using an Affymetrix labeling kit and hybridized to the genome tiling array.
(961 KB EPS)

Table S1 Oligonucleotides (55 KB DOC)

## Abbreviations

LTR: long terminal repeat
RT-PCR: reverse-transcriptase PCR
SREBP: sterol regulatory element binding protein
