# Supplementary material for: SREBP Controls Oxygen-Dependent Mobilization of Retrotransposons in Fission Yeast
Source: PLoS Genet. 2007 Aug 10;3(8):e131. doi: 10.1371/journal.pgen.0030131 (PMC1941750; doi:10.1371/journal.pgen.0030131)
Supplement: Supplementary file 1 [file pgen.0030131.sg001.doc]

Sehgal_Supplemental Figure 1


Tf2-3     1 TGTCAGCAATACTACACTACGCTATAATACACTACGTTGAGTATCACTATATGTC
Tf2-11    1 TGCGTAGCTGTCA-------GCAATACTGCACTACGTTGCGTATCACTATATGTC
                               
                                SRE
Tf2-3    56 ACATGTTCTAATTATATATCGTACCATGTATGATACGATATGGAGATTGATCTTA
Tf2-11   49 ACATGTTC-AACATCATATCGTAGATA--------TCCG-------------TTG

                                                TATA
Tf2-3   111 ATGATAATCTATTAAGATCAATATTATCTGAATACTATAAATAGAGCTACTGCTG
Tf2-11   82 ATAACAAG-TATTTAGA-------------AATATTATAAATAGCGTTACAACTG


Tf2-3   166 AACCTCGTTCCTCAGTTCAGTTATGAGCTATATTAGTGATAGGTAACATTATAAC 
Tf2-11  123 AACCTCGTTCCTCAGTTCAGTTATGAGCTATATTATTGATAGGTAACATTATAAC


Tf2-3   221 CCAGTTAATACAATACCTATACTCAGTTGCTACTTATACAACCTGTGTATCGTAA
Tf2-11  178 CCAGTTAATACAATACCTATACTCAGTTGCTTCTCATACAACCTGTGTATTGTAA


Tf2-3   276 TATAATAGATCACAAGGAAAACTCACCGCAGTTCTACGTATCCTTAAATCAGATA
Tf2-11  243 TATAATAGATCACAAGGAAAACTCACCACAGTTCTACGTATCCTTAAATCAGATA


Tf2-3   331 CCAAACTGCGTAGCTTACA
Tf2-11  288 CCAAACTGCGTAGCTTACA
