# Supplementary material for: SREBP Controls Oxygen-Dependent Mobilization of Retrotransposons in Fission Yeast
Source: PLoS Genet. 2007 Aug 10;3(8):e131. doi: 10.1371/journal.pgen.0030131 (PMC1941750; doi:10.1371/journal.pgen.0030131)
Supplement: Supplementary file 2 [file pgen.0030131.st001.doc]

**Sehgal_Supplemental Table I**

**Oligonucleotides**

| **Knock-in of *ura4^+^* to Tag Different Transposons** | |
| --- | --- |
| Forward | ^5’^ CTCCTTCTGGGTTCAAAGGAGAAAGAACTTTGGAAAGACTGTTA  TCCCTTTTGAAATCTCCCAAAAGGGAAACATATACACGCCAGGGT  TTTCCCAGTCACGAC^3’^ |
| Reverse | ^5’^GATGTTTGAATTCCATCCAAATTCTCTGGTCTCCATCGTTTTGCT  CTTGCTTTCATATAACGATAATTTGCGTAGGACATAGCGGATAAC  AATTTCACACAGGA^3’^ |
|  |  |
| **Oligos for Gel Shift** | |
| Tf2 forward | ^5’^ATGGCCATATCGTACCAT^3’^ |
| Tf2 reverse | ^5’^ATGGCCATATGGTACGAT^3’^ |
| Tf2-11 forward | ^5’^ATGGCCATATCGTAGATA^3’^ |
| Tf2-11 reverse | ^5’^ATGGCCATTATCTACGAT^3’^ |
|  | Underlined sequence is the non-specific DNA used for fill-in reaction. |
|  |  |
| **Oligos for ChIP** | |
| Tf2-3 forward | ^5’^GTATAAGGCTCCTAGTAACTTAG^3’^ |
| Tf2-4 forward | ^5’^GCGCATCTTTCATGGGTAGT^3’^ |
| Tf2-11 forward | ^5’^GACCAATTCTAACCCTTCAA^3’^ |
| Reverse | ^5’^ATAGCTCATAACTGAACTGA^3’^ |
|  |  |
| **For Tiling Array** | |
| Random primer with 5’ adapter | ^5’^CGCGCCAATACGTCGTGGANNNNNNNNN^3’^ |
| LTR specific I | ^5’^CAGATACCAAACTGCGTA^3’^ |
| LTR specific II | ^5’^CACCGCAGTTCTACGTATCC^3’^ |
|  |  |
| **For *SPCC11E10.07C*** | |
| Primer A | ^5’^TCAGTTCAGTTATGAGCTAT^3’^ |
| Primer B | ^5’^ATGGTGGAATCGGATTCTTC^3’^ |
| Primer C | ^5’^ACAGCCTGAGCCGCTAGGTC^3’^ |
| **For *SPAC2E1P3.02c*** | |
| Primer A | ^5’^TCAGTTCAGTTATGAGCTAT^3’^ |
| Primer B | ^5’^ATGCTTAACGAGCCGAATGC^3’^ |
| Primer C | ^5’^GGTTCATCCCTCCTTGCCAC^3’^ |
|  |  |
| **Oligos for Inverse PCR, present in neomycin** | |
| Forward | ^5’^CTCATAACACCCCTTGGTACCGG^3’^ |
| Reverse | ^5’^CCATATTCAACGGGAAACGTCTTGC^3’^ |
|  |  |
| **Oligos for vectorette PCR** | |
| With EcoRI linker | ^5’^AATTGAAGGAGAGGACGCTGTCTGTCGAAGGTAAGGAACGGA  CGAGAGAAGGGAGAG ^3’^ |
| With SpeI linker | ^5’^CTAGGAAGGAGAGGACGCTGTCTGTCGAAGGTAAGGAACGGA  CGAGAGAAGGGAGAG^3’^ |
| Common annealing | ^5’^CTCTCCCTTCTCGAATCGTAACCGTTCGTACGAGAATCGCTGTC  CTCTCCTTC^3’^ |
| For PCR | ^5’^CTCTCCCTTCTCGAATCGTAA^3’^ |
